# The Role of Affect Spin in the Relationships between Proactive Personality, Career Indecision, and Career Maturity

**DOI:** 10.3389/fpsyg.2015.01754

**Published:** 2015-11-18

**Authors:** In-Jo Park

**Affiliations:** Department of Psychology, Yonsei UniversitySeoul, South Korea

**Keywords:** proactive personality, affect spin, career indecision, career maturity, day reconstruction method (DRM)

## Abstract

This study attempted to investigate the influence of proactive personality on career indecision and career maturity, and to examine the moderating effects of affect spin. The author administered proactive personality, career indecision, and career maturity scales to 70 college students. Affect spin was calculated using the day reconstruction method, wherein participants evaluated their affective experiences by using 20 affective terms at the same time each day for 21 consecutive days. Hierarchical regression analyses showed that proactive personality significantly predicted career indecision and career maturity, even after controlling for valence and activation variability, neuroticism, age, and gender. Furthermore, affect spin moderated the associations of proactive personality with career indecision and maturity. The theoretical and practical implications of the moderating effects of affect spin are discussed.

## Introduction

Individuals are influenced by their emotions in many ways. Emotions are ubiquitous, involving neurological, physiological, and behavioral changes that facilitate adaptation, assist decision-making, and increase happiness (Frijda, 2008; Hartung, 2011). Furthermore, they are linked to cognitive systems involved in decision-making and can improve decision making (Emmerling and Cherniss, 2003). Kidd (1998, 2004) highlighted the role of affect in career decision-making and development and argued that emotion can help expand career theory. What aspects of emotion are related to career decisions?

The role of emotion in careers has been studied in relation to anxiety, emotional intelligence, and affective variability. Anxiety is associated with career indecision and indecisiveness (Fuqua et al., 1987; Kaplan and Brown, 1987; Gati et al., 2010). Brown et al. (2003) showed that emotional intelligence is related to career decision-making self-efficacy and career commitment. Increasing emotional intelligence through training programs can reduce career indecisiveness and difficulties in decision making (Di Fabio and Kenny, 2011). Regarding affective variability, Hirschi and Freund (2014) found that positive emotions predicted the level of career engagement over a 13-week period in a longitudinal study. Jung et al. (2015) examined the moderating effects of *affect spin*, “the standard deviation across time of the angles of the vectors described by the individual's core affect space positions” (Kuppens et al., 2007), on future time perspective and career decisions, using the experience sampling method.

The present study focuses on the role of affect spin in the relationships between proactive personality and career decision-making processes in order to address the following question: Are the relationships between proactive personality and career decision-making processes weaker or stronger depending on the level of affect spin? This question is highly appropriate because affect spin might influence the relationship between proactive personality and career decision-making processes. Generally, compared with non-proactive people, proactive people more actively search their perspective job and are more motivated in their work, which are associated with less career indecision and more career maturity (Hsieh and Huang, 2014). However, this relationship could be moderated by affective experience, whereby high fluctuation in affective experience—namely, high affect spin—would influence the relationship between proactive personality and career indecision or career maturity.

Affect spin has been shown to influence the relationship between characteristics subject to individual differences (e.g., future time perspective) and career decision-making processes (Jung et al., 2015). Zimbardo et al. (1997) divided time perspective into six zones: past-negative, past-positive, present-fatalistic, present-hedonistic, future, and transcendental-future. Specifically, affect spin moderates the relationships between future time perspective—a cognitive unit that is flexible and differs across individuals—and career decision-making self-efficacy and career choice anxiety by reducing the facilitative effect of future time perspective. Among these six zones, the future zone is considered a flexible cognitive unit that is sensitive to individual differences (Marko and Savickas, 1998). However, there is currently no research on the role of affect spin in the relationship between trait-like predictor variables and career decision-making processes, even though such research would be important for career decision-making. Similar to Jung et al.'s study, studies that examine the role of affect spin on the relationship between trait-like predictor variables—such as proactive personality—and career decision processes would extend current knowledge of career decision-making. Thus, the current study investigates the role of affect spin in the relationship between proactive personality and career decision-making processes, including career indecision and career maturity.

## Theoretical background

### Affective-state theories: Basic emotion theory and dimension theory of emotion

Theoretical perspectives on human affective states fall under the purview of basic emotion theory or the dimension theory of emotion. The former is concerned with identifying fundamental emotions and their relations with secondary ones (Ekman and Davidson, 1994). For instance, Izard (2007) suggests the existence of six discrete basic emotions: interest, joy/happiness, sadness, anger, disgust, and fear. These theorists (e.g., Izard, 1993; Ekman, 1999) assume that basic emotions are outcomes of environmental adaptation and have distinctive facial units, physiology, and related behavioral tendencies.

**Figure 1:**
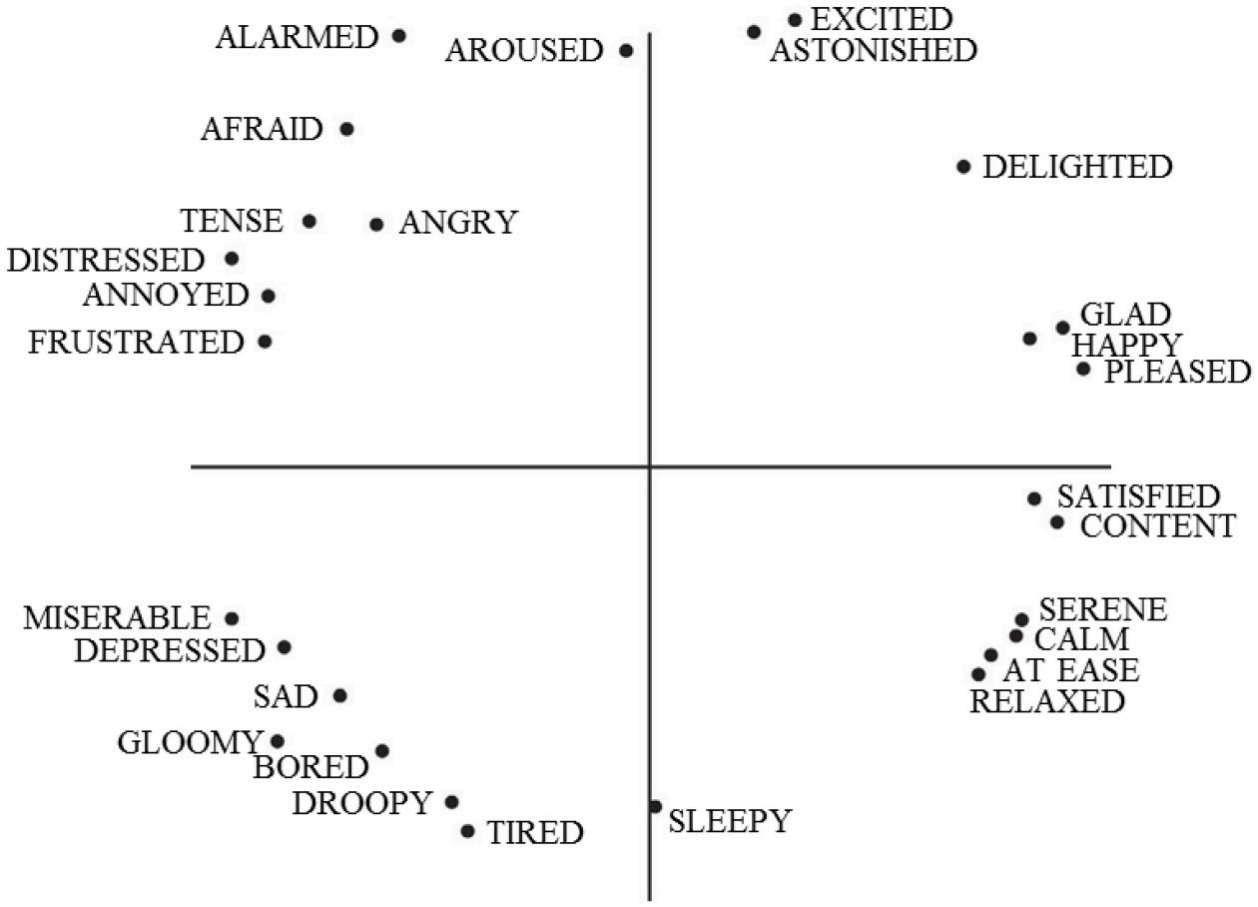
**The circumplex model of affect**. From “A circumplex model of affect” by Russell (1980).

The dimension theory regards emotions as composites of several dimensions (Park and Min, 2005). Dimensional theorists argue that emotions are points in a continuous space rather than distinct units. As shown in Figure 1, Russell (1980) arranged emotional words along horizontal and vertical dimensions. The horizontal dimension is the circumplex space of pleasure–displeasure, while the vertical dimension is that of activation–deactivation. Russell's circumplex model of affect is supported by numerous cross-cultural studies in various languages (Park and Min, 2005).

### Intraindividual variability in affect

Individuals generally show stable and reliable affective fluctuations, the extent of which is subject to individual differences (Larsen, 1987). In other words, *intraindividual variability in affect*—defined as short-term fluctuations in the degree of variation and time-based dynamics of affective states (Ram and Gerstorf, 2009; Röcke and Brose, 2013)—is stable across time (Larsen, 1987; Penner et al., 1994; Eaton and Funder, 2001; Kuppens et al., 2007).

**Figure 2:**
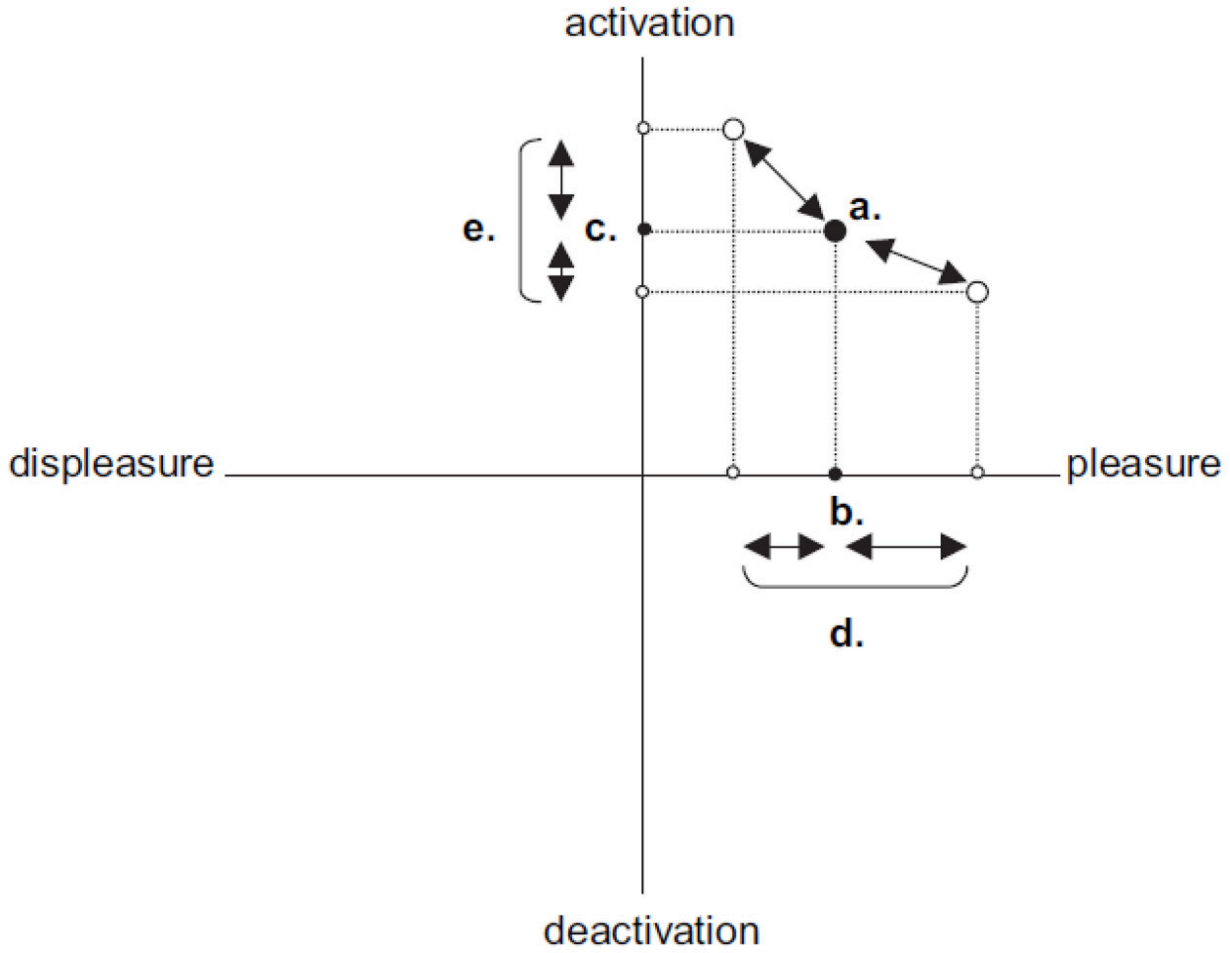
**Graphical example of valence and activation variability**. From “Individual differences in core affect variability and their relationship to personality and psychological adjustment” by Kuppens et al. (2007).

The dimension theory of emotion proposes two types of intraindividual variability: *valence* and *activation*. These are within-person standard deviations across Cartesian coordinates based on the circumplex model (Kuppens et al., 2007). In the circumplex model, valence variability is calculated using the horizontal pleasure–displeasure line and activation variability is calculated using the vertical activation–deactivation line. Valence variability denotes the extent to which an individual varies in terms of pleasure states, while activation variability reflects the extent to which the emotion is activated. Figure 2 depicts valence and activation variability such that the “d” Cartesian coordinates represent valence variability and “e” coordinates represent activation variability.

**Figure 3:**
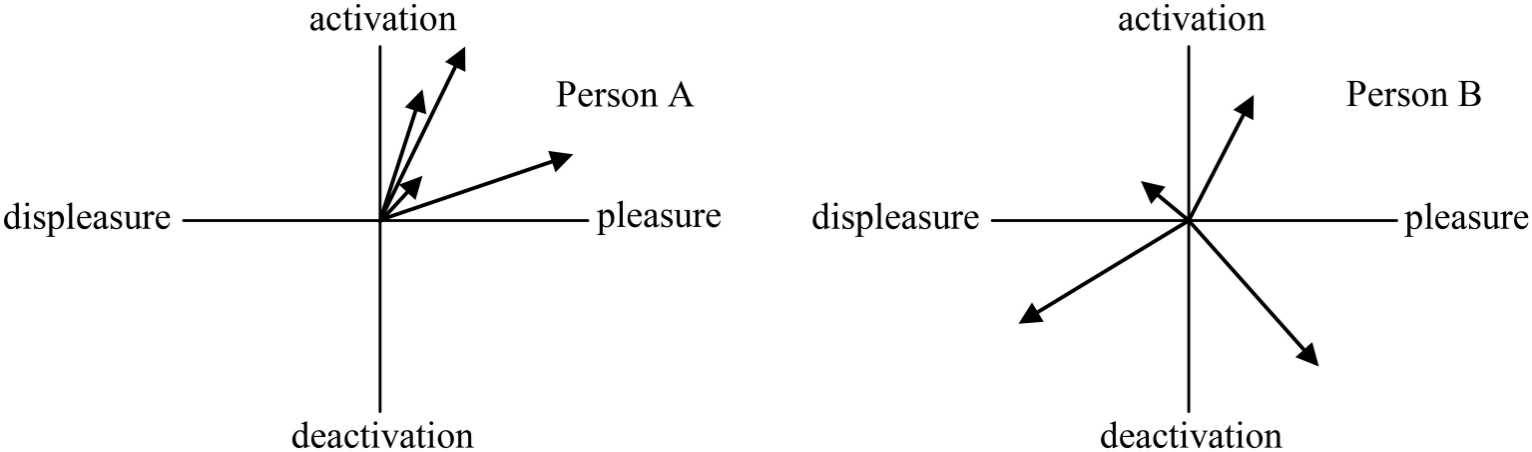
**Illustration of affect spin using two hypothetical persons**.

Recently, there has been a growing interest in other variables showing intraindividual variability such as affect spin (e.g., Kuppens et al., 2007; Jung et al., 2015). Affect spin is regarded as shifts in the quality of an individual's core affect—namely, the trait variability of an individual's affective states (Beal et al., 2013). It reflects the extent to which an individual varies within the core affect space, meaning that it represents how individuals shift between qualitatively different affective states without consideration of intensity of feeling (Kuppens et al., 2007). For instance, in Figure 3, although Persons A and B experience the same intensity of feeling, Person B has greater affect spin because that person displays greater variability in the quality of his/her affect.

Affect spin can provide a more comprehensive evaluation of affective variability compared to other unidimensional measures and is therefore considered the most representative index of intraindividual affective changes (Beal et al., 2013).

### Diary methods for assessing affective states

A repeated measures design is typically used to measure intraindividual variability. Data from daily life provides ecological validity that is difficult to obtain from a single-administration survey (Reis and Judd, 2000). Two primary methods have been used to assess affective state with repeated measures: *experience sampling method* (ESM) and *day reconstruction method* (DRM).

The ESM assesses individuals' affective states in natural settings at specific times (Csikszentmihalyi and Larson, 1987; Stone and Shiffman, 1992). For example, after receiving an alert via text or personal digital assistant, participants rate their current affect. Prior studies involved rating one's affect 4–9 times daily for several consecutive days (e.g., Kuppens et al., 2007; Beal et al., 2013; Jung et al., 2015). However, this method has a limitation in calculating affect spin in that it is done over a relatively short period, which can bias assessment of affect spin. In other words, if an individual experienced several positive and negative events that elicited emotions, he or she would show greater affect spin compared to an individual who had experienced relatively few events. Alternatively, the DRM (Kahneman et al., 2004) is also employed in research on affect (Stone et al., 2006) and requires participants to recall events or emotions. Using the DRM, Kuppens et al. (2007) conducted a study where participants reflected on their affect once daily (just before going to bed) for 2 weeks. This method reduces participants' burden of needing to provide multiple ratings daily and avoids bias resulting from a relatively short evaluation period.

### Proactive personality, career indecision, career maturity, and affect spin

People's personalities differ considerably in many aspects. A proactive personality is marked by “a relatively stable tendency to effect environmental change” (Bateman and Crant, 1993). Individuals with a highly proactive personality are more motivated to attain planned goals compared to those with a less proactive personality (Parker et al., 2010). Proactive personality may therefore facilitate motivational processes and produce better outcomes for individual (Parker et al., 2010; Lin et al., 2014). Indeed, motivated information processing facilitates decision-making (De Dreu et al., 2008), which in turn can reduce career indecision.

The current study focuses on the fact that a highly proactive personality implies a tendency to actively make appropriate career decisions and improve career attitude. Previous research has shown that proactive personality is positively associated with career decision-making self-efficacy and job search self-efficacy (Hsieh and Huang, 2014). Thus, proactive personality, mediated by domain-specific self-efficacy, may influence people's behavior and outcomes (Frese and Fay, 2001; Brown et al., 2006). The findings suggest that individuals who are more proactive make career decisions confidently, thereby avoiding career indecision, which indirectly suggests a negative association between proactive personality and career indecision. The present study therefore hypothesized the following:

**Hypothesis 1**. Proactive personality will be negatively associated with career indecision.

This study's primary aim was to examine the role of affect spin in the relationship between proactive personality and career decision-making processes. Affect spin is associated with psychological and behavioral maladjustments such as depression, life dissatisfaction, and fatigue (Kuppens et al., 2007; Beal and Ghandour, 2011). For instance, Hong et al. (2012) empirically demonstrated that affect spin is negatively related to life satisfaction among employees, which would suggest that affect spin negatively influences the relationship between proactive personality and career decision-making. This is similar to Jung et al.'s (2015) study, wherein affect spin weakened the relationship between future time perspective and career decisions.

Individuals with high affect spin experience unpredictable affective states (Beal et al., 2013). Unpredictability increases uncertainty when planning actions (Frese and Zapf, 1994). Moreover, individuals with high affect spin are sensitive to both positive and negative events (Beal and Ghandour, 2011) and require more cognitive resources to cope with such events, potentially leading to fatigue. Furthermore, difficulties in career decision-making may increase with increased uncertainty and fatigue. Anxiety and neuroticism are also positively associated with career indecision (Saka and Gati, 2007), and since neuroticism is positively related to affect spin, it may be that affect spin weakens the facilitative effect of proactive personality on decision-making, resulting in greater career indecision. Thus, individuals with high affect spin may demonstrate high career indecision even when they have highly proactive personalities. The author therefore hypothesized the following:

**Hypothesis 2**. Affect spin will moderate the negative relationship between proactive personality and career indecision, such that the relationship will be weaker in individuals with high affect spin compared to those with low affect spin.

Super (1990) defines career maturity as an individual's readiness to deal with the developmental missions of one's life stage (Janeiro, 2010). Super's concept of career maturity comprises two dimensions: attitudes toward and competencies for developing one's career (Savickas et al., 2002). The former includes attitudes toward career planning and exploration; and the latter comprises decision-making competence and accumulation of occupational information.

Career exploration has been found to increase career maturity (Super, 1983; Watson and Stead, 1997). It is also related to various personality characteristics, particularly extraversion (Savickas et al., 2002). Being proactive is similar to being extraverted in that it implies being motivated, careful, organized, and goal directed (Fay and Frese, 2000; Grant and Ashford, 2008; Bindl and Parker, 2009; Parker et al., 2010). The author expects that highly proactive individuals would have highly positive attitudes and would be interested in developing their competencies; these qualities reflect career maturity, which in turn helps in career decision-making. Therefore, the author hypothesized the following:

**Hypothesis 3**. Proactive personality will be positively associated with career maturity.

Individuals with high affect spin appear to show instability in interpersonal relationships (Timmermans et al., 2010), often fail to obtain positive feedback from others (Côté et al., 2006), and are at risk for depression (Kuppens et al., 2007). They may have lower career self-efficacy compared to those with low affect spin. Given that high affect spin inhibits career-related self-efficacy, it may also weaken the positive influence of proactive personality on career maturity. Thus, individuals with high affect spin may have lower career maturity because of negative feedback and depression, even if they are highly proactive. The author therefore hypothesized the following:

**Hypothesis 4**. Affect spin will moderate the positive relationship between proactive personality and career maturity, such that the relationship will be weaker in individuals with high affect spin compared to those with low affect spin.

## Method

### Participants

In the present study, the sample size was decided based on Kuppens et al. (2012) study using the ESM, in which 79 participants rated their affect for 14 consecutive days. The present sample comprised 70 college students enrolled in the learning psychology course at a college. Their participation was in fulfillment of class requirements; they could receive course credits for completing the study (i.e., rating their daily affective states for 21 consecutive days and completing the final survey). Since nine students withdrew from the study, data from 61 students (21 male and 40 female; mean age: 21.2 years) were analyzed.

### Procedure

Participants first attended an introductory session where the author provided basic knowledge about affect and the procedure for rating their affective states on the website every day for 21 consecutive days using their personal computer or smartphone. They were instructed to evaluate their affect at around 10 p.m., when they would receive a daily reminder text message, for 3 weeks. If they missed a rating, they were instructed to not compensate for it on the following day. Participants were informed that the time taken to respond would be recorded in order to motivate them to follow the instructions closely.

On the first day, participants received a text message in the middle of the day that read, “Today is the first day of rating your affective experience; this will continue for the following 21 days. You will receive text messages at 10 p.m.” The daily text message included a short instruction, such as “Please report the affect that you experienced today using your computer or smartphone.” At the end of the study period, participants were given the final survey, which included the proactive personality, career indecision, and career maturity scales. Data on 1192 days' responses from 61 participants were obtained (*M* = 19.54, *SD* = 1.22). This study was conducted in accordance with the Declaration of Helsinki and was approved by Gyeongsang National University's review board. All participants gave written informed consent in accordance with the Declaration of Helsinki.

### Measures

#### Repeated assessment of core affect

Kuppens et al. (2007) measured daily affect based on the four quadrants of the core affect space used to calculate valence variability, activation variability, and affect spin: positive active affect (pa), positive deactive affect (pd), negative active affect (na), and negative deactive affect (nd). Participants' affective experiences were classified as “pa,” for enthusiastic, happy, alert, proud, and excited affective terms; “pd” for the terms calm, peaceful, satisfied, relaxed, and content; “na” for nervous, embarrassed, upset, stressed, and tense; and “nd” for sluggish, sad, bored, depressed, and disappointed. In the current study, the 20 affective terms were translated into Korean. Participants rated each term on a 7-point Likert type scale ranging from 1 (did not feel this way at all) to 7 (felt this way strongly) according to the instructions, “Indicate in the space next to each term how strongly you felt that way today.”

#### Proactive personality

Proactive personality was measured with the short Proactive Personality Scale, which comprises 10 items (Seibert et al., 1999). Bateman and Crant (1993) initially developed and validated a 17-item Proactive Personality Scale, which Seibert et al. (1999) subsequently shortened to 10 items. Hwang and Tak (2010) translated the short Proactive Personality Scale from English into Korean for their study. An example item is, “Wherever I have been, I have been a powerful force for constructive change.” The response format was a 7-point scale, ranging from 1 (strongly disagree) to 7 (strongly agree). Cronbach's alpha was 0.84 in this study.

#### Career indecision

Participants' career indecision was evaluated using the corresponding subscale of the Career Decision Scale (CDS), developed and validated by Osipow et al. (1976). Ko (1992) translated the 18-item career indecision subscale into Korean for her study. An example item is, “I need more information about what different occupations are like before I can make a career decision.” Items were rated on 5-point scale, ranging from 1 (like me) to 5 (not like me). Higher scores reflect greater indecision. Cronbach's alpha for the scale in this study was 0.90.

#### Career maturity

Participants' career maturity was assessed with the Korean version of the Career Maturity Inventory's (CMI) Attitude Scale, which comprises 47 items. This scale was validated by Kim (1997). The original CMI comprised 75 dichotomous items (“agree/true” and “disagree/false”; Crites, 1978) but was later revised and shortened to 50 items (Crites and Savickas, 1996). Kim's version (1997) uses a 4-point scale based on that of the original CMI that ranges from 1 (not like me) to 4 (like me). Higher scores represent more highly developed attitudes toward career development and are associated with vocational decidedness (Fuqua and Newman, 1989). Cronbach's alpha was 0.90 in the present study.

#### Control variables

Neuroticism was controlled for since it has been linked to career indecision (e.g., Chartrand et al., 1993). Neuroticism was measured with the 10 Big Five aspects of the International Personality Item Pool (IPIP) validated by Goldberg (2001). This study also controlled for valence variability, activation variability, age, and gender.

### Data analysis

The present study followed the procedure used in Kuppens et al.'s (2007) Study 2 to calculate affect spin. First, data from daily ratings for pa, pd, na, and nd items were averaged to calculate a daily pa, pd, na, and nd score for each participant. Using these scores, valence [(pa + pd) – (na + nd)] and activation [(pa + na) – (pd + nd)] scores were produced for each evaluation occasion. Additionally, valence variability (within-person standard deviation of valence) and activation variability (within-person standard deviation of activation) were calculated for each participant.

Second, the author employed the following steps based on prior studies (Kuppens et al., 2007; Jung et al., 2015). At each time point “*t*,” the unit vector was calculated by transforming the observed vectors for each of the participants' evaluations, as follows:

$$\text{Unit Vector}=\frac{{\text{valence}}_{\text{t}}}{\sqrt{{\text{valence}}_{\text{t}}^{2}{+\text{activation}}_{\text{t}}^{2}}}\;\frac{{\text{activation}}_{\text{t}}}{\sqrt{{\text{valence}}_{\text{t}}^{2}{+\text{activation}}_{\text{t}}^{2}}}$$

Then, the resultant vector (R) for all evaluations for one participant was computed by summing all observations.

$$\text{R}=\sum_{t=1}^{n}\frac{{\text{valence}}_{t}}{\sqrt{{\text{valence}}_{\text{t}}^{2}+{\text{activation}}_{\text{t}}^{2}}}\;\sum_{t=1}^{n}\frac{{\text{activation}}_{t}}{\sqrt{{\text{valence}}_{\text{t}}^{2}+{\text{activation}}_{\text{t}}^{2}}}$$

Subsequently, the length of R was normalized by dividing by the participants' total number of evaluations.

$$\begin{array}{l} \;\frac{\left\|\vec{R}\right\|}{n}\\ =\frac{\sqrt{{\left(\sum_{t=1}^{n}\frac{{\text{valence}}_{t}}{\sqrt{{\text{valence}}_{t}^{2}+{\text{activation}}_{t}^{2}}}\right)}^{2}+{\left(\sum_{t=1}^{n}\frac{{\text{activation}}_{t}}{\sqrt{{\text{valence}}_{t}^{2}+{\text{activation}}_{t}^{2}}}\right)}^{2}}}{n} \end{array}$$

Finally, the standard deviations of the angles of unit vectors—representing affect spin—were calculated.

$$\text{Affect Spin}=\sqrt{-2\ln\left(\frac{\left\|\vec{R}\right\|}{n}\right)}$$

A hierarchical regression analysis was employed to test the hypotheses (Cohen and Cohen, 1983). The current study included neuroticism, valence variability, activation variability, age, and gender as control variables in the first step. In the second step, proactive personality, affect spin, and the product of proactive personality and affect spin were entered as predictor, moderator, and interaction term, respectively. This study used mean-centered data for proactive personality and affect spin to reduce multicollinearity (Jaccard et al., 1990). Following Aiken et al. (1991), the simple slope test was conducted to examine the direction of the interactions.

## Results

### Descriptive statistics and correlations

The means, standard deviations, and intercorrelations of variables are presented in Table 1. With regard to the main variables, the results showed that proactive personality was positively associated with career maturity (*r* = 0.44, *p* < 0.01) and negatively associated with career indecision (*r* = −0.43, *p* < 0.01). Further, career indecision was negatively associated with career maturity (*r* = −0.85, *p* < 0.01). These results supported the positive effect of proactive personality on career maturity and its negative effect on career indecision.

**Table 1:** **Means, standard deviations, and correlations among study variables (***N*** = 70)**.

	***M***	***SD***	**1**	**2**	**3**	**4**	**5**	**6**	**7**	**8**
1. Age	21.20	1.73	–							
2. Gender^a^	1.68	0.47	−0.45^**^	–						
3. VV	2.04	0.60	0.11	−0.10	–					
4. AV	1.58	0.57	0.05	0.13	0.47^**^	–				
5. Neuroticism	3.18	0.81	−0.06	0.23	0.15	0.14	–			
6. Affect spin	1.33	0.44	−0.19	0.11	0.16	0.17	0.14	–		
7. PP	4.16	0.82	0.25	−0.10	−0.02	0.04	0.06	−0.09	–	
8. CI	2.52	0.50	−0.14	0.26^*^	−0.12	−0.05	0.17	0.26	−0.43^**^	–
9. CM	2.68	0.33	0.06	−0.09	0.27^*^	0.09	−0.13	−0.24	0.44^**^	−0.85^**^

* *p < 0.05*,

** *p < 0.01*.

a *1, male; 2, female; VV, valence variability; AV, activation variability; PP, proactive personality; CI, career indecision; CM, career maturity*.

With reference to the control variables, valence variability was positively associated with activation variability (*r* = 0.47, *p* < 0.01) and career maturity (*r* = 0.27, *p* < 0.05). Gender was positively associated with career indecision (*r* = 0.26, *p* < 0.05) and was negatively associated with age (*r* = −0.45, *p* < 0.01). Neuroticism—as a control variable—was not significantly related to the outcome variables.

### Predictor and moderator effects

To examine the hypotheses, hierarchical regression analysis was conducted. Neuroticism, valence variability, activation variability, and demographic variables such as age and gender were entered in Step 1 (see Table 2). Proactive personality and affect spin were mean-centered to avoid multicollinearity (Jaccard et al., 1990); then, the product of proactive personality and affect spin was derived. The mean-centered proactive personality, mean-centered affect spin, and the product of proactive personality and affect spin were entered in Step 2.

**Table 2:** **Results of hierarchical regression analyses predicting career indecision and career maturity**.

	**Career indecision**		**Career maturity**	
	**Model 1-1**	**Model 1-2**	**Model 2-1**	**Model 2-2**
**STEP 1**				
Age	0.04	0.20	−0.03	−0.22
Gender	0.33^*^	0.33^*^	−0.13	−0.14
Neuroticism	0.10	0.15	−0.13	−0.18
Valence variability	−0.12	−0.14	0.23	0.32^*^
Activation variability	0.02	0.03	0.00	0.01
**STEP 2**				
Proactive personality (A)		−0.44^**^		0.47^***^
Affect spin (B)		0.22		−0.26^*^
A × B		0.25^*^		−0.23^*^
*R*^2^	0.13	0.43	0.09	0.42
Adjusted *R*^2^	0.04	0.32	0.01	0.32
*F*	1.50	4.09^**^	0.93	4.05^**^
Δ*R*^2^	0.13	0.29	0.09	0.33
*^a^F*	1.50	7.48^**^	0.93	8.53^***^

* *p < 0.05*,

** *p < 0.01*,

*** *p < 0.001*.

a *F refers to changes from the prior model*.

Hypothesis 1 stated that proactive personality would be negatively associated with career indecision. The regression analysis showed that proactive personality, affect spin, and their product in Model 1-2 accounted for 29% of the variance in career indecision (*p* < 0.01). After controlling for neuroticism, valence variability, activation variability, age, and gender, proactive personality significantly and negatively predicted career indecision in Model 1-2 (β = −0.44, *p* < 0.01), thus supporting Hypothesis 1.

**Figure 4:**
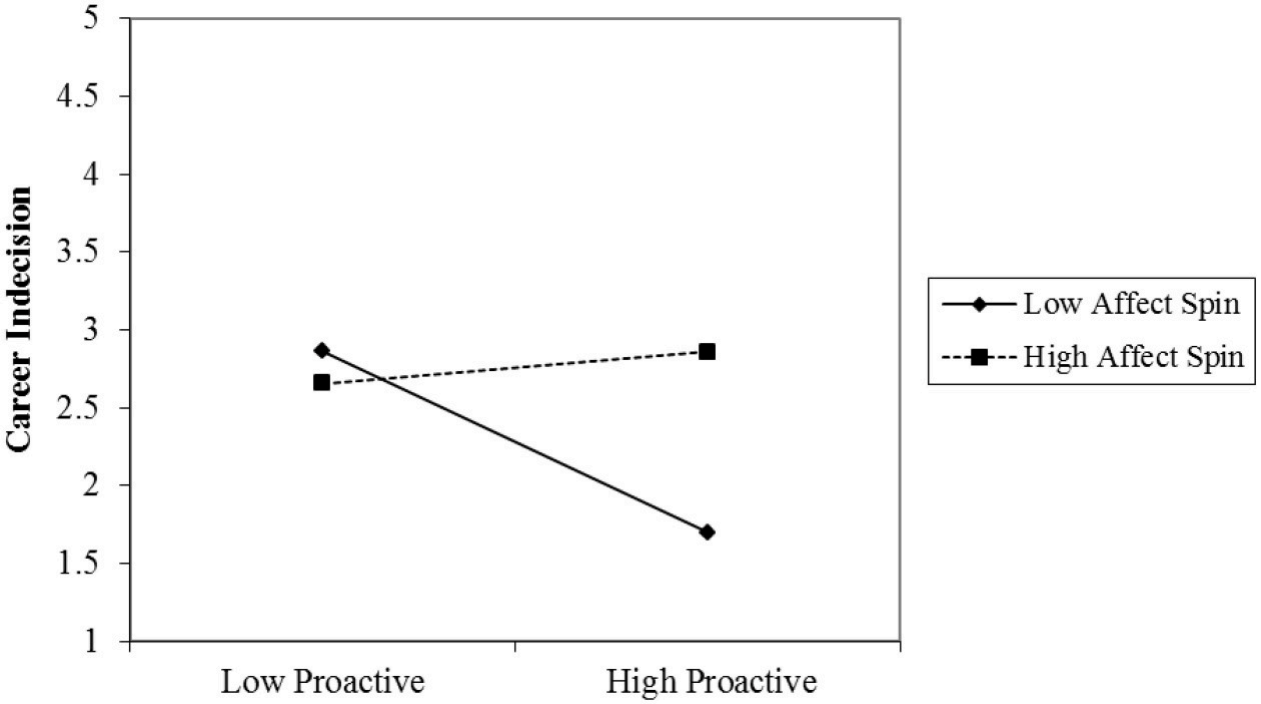
**The moderating effect of affect spin in the relationship between proactive personality and career indecision**.

Hypothesis 2 proposed that affect spin would moderate the negative relationship between proactive personality and career indecision, such that the relationship would be weaker in individuals with high affect spin compared to those with low affect spin. Model 1-2 showed that the product of proactive personality and affect spin significantly predicted career indecision (β = 0.25, *p* < 0.05), thus supporting Hypothesis 2. A simple slope analysis was conducted to confirm the results (Aiken et al., 1991); when affect spin was higher, the relationship between proactive personality and career indecision was weaker (*B* = −0.09, *SE* = 0.12, *t* = −0.71, *p* > 0.05), and when proactive personality was lower, the relationship was stronger (*B* = −0.39, *SE* = 0.12, *t* = −3.22, *p* < 0.01; see Figure 4).

Hypothesis 3 proposed that proactive personality would be positively associated with career maturity. The regression analysis indicated that proactive personality, affect spin, and their product in Model 2-2 accounted for 33% of the variance in career indecision (*p* < 0.01). The results showed that proactive personality significantly and positively predicted career maturity in Model 2-2 (β = 0.47, *p* < 0.001), thus supporting Hypothesis 3. Additionally, affect spin—the moderator—significantly and negatively predicted career maturity (β = −0.26, *p* < 0.05), indicating that individuals with higher affect spin showed lower career maturity.

**Figure 5:**
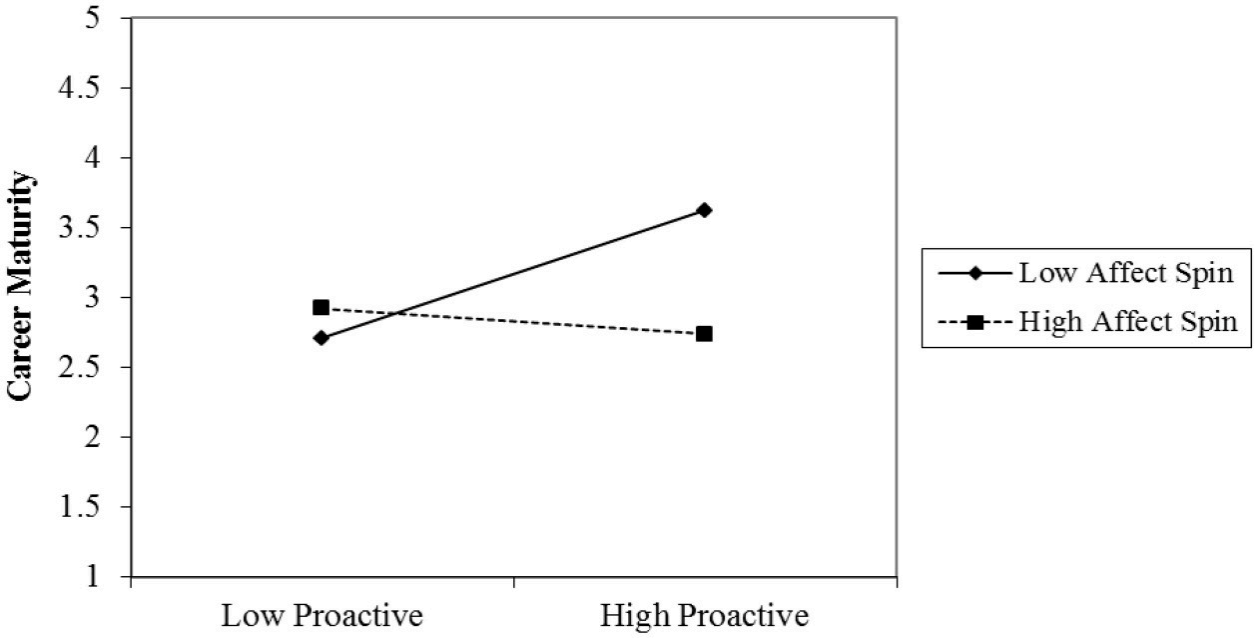
**The moderating effect of affect spin in the relationship between proactive personality and career maturity**.

Hypothesis 4 proposed that affect spin would moderate the positive relationship between proactive personality and career maturity, such that the relationship would be weaker in individuals with high affect spin relative to those with low affect spin. Model 2-2 revealed that the product of proactive personality and affect spin significantly predicted career indecision (β = −0.23, *p* < 0.05), thus supporting Hypothesis 4. The results of the simple slope analysis showed that when affect spin was higher, the relationship between proactive personality and career maturity was weaker (*B* = 0.06, *SE* = 0.08, *t* = 0.76, *p* > 0.05), and when affective spin was lower, the relationship was stronger (*B* = 0.26, *SE* = 0.08, *t* = 3.26, *p* < 0.01; see Figure 5).

## Discussion

The present study examined the relationships between proactive personality and career decision-making processes and the moderating effects of affect spin on these relationships. DRM was used to assess affect spin. Participants described their daily affective states for 21 days using 20 affective terms. The results showed that proactive personality was significantly related to career indecision and career maturity. Furthermore, affect spin significantly moderated the relationship between proactive personality and career indecision, and consequently, career maturity.

As expected, proactive personality negatively influenced career indecision. According to prior research (e.g., Brown et al., 2006; Hsieh and Huang, 2014), proactive personality influences career decision-making processes such as career decision-making self-efficacy and job search self-efficacy. The present study extends these findings, as proactive personality was negatively associated with career indecision, suggesting that proactive personality plays an important role because it enhances confidence in career decision-making and job search, and reduces career indecision.

The present study found support for Hypothesis 2: affect spin moderated the negative relationship between proactive personality and career indecision. Given that proactive personality is a persistent personality characteristic like the Big Five, investigating a state-like factor (i.e., affect spin) that can work as a moderator is valuable. The results showed that high affect spin is likely to have an inhibitory effect on proactive individuals, weakening the offsetting effect of proactive personality on career indecision. According to Beal and Ghandour (2011), high affect spin is related to neuroticism, typically resulting in negative interpretations, perceptions, and reactions to information (Baumeister et al., 2001; Rozin and Royzman, 2001; Jung et al., 2015). Individuals with high affect spin may have difficulty with career decisions despite being highly proactive.

Hypothesis 3, which proposed that proactive personality would be positively related to career maturity, was supported by the results. Compared to less proactive individuals, highly proactive individuals are more likely to engage in career exploration (Brown et al., 2006), which in turn leads to career maturity (Super, 1983). Moreover, being proactive implies a tendency to be motivated, alert, organized, and goal directed (Frese and Fay, 2001; Grant and Ashford, 2008; Bindl and Parker, 2009; Parker et al., 2010). Career maturity is positively related to career exploration and planning (Savickas et al., 2002). These are also associated with proactive personality. Thus, the results suggest that proactive personality may positively influence career maturity because of its link to career planning and exploration.

Hypothesis 4, which proposed that affect spin would moderate the positive relationship between proactive personality and career maturity, was also supported. The results showed that the relationship between proactive personality and career maturity was weaker in individuals with high affect spin, compared to those with low affect spin. Those results correspond to those of Jung et al.'s study (2015), in which the relationship between future time perspective and career decision-making self-efficacy was weaker in students with high affect spin relative to those with low affect spin. High variability in core affect (i.e., high affect spin) appears to be positively related to optimism, conscientiousness, and extraversion, but negatively associated with neuroticism and pessimism (Kuppens et al., 2007; Beal and Ghandour, 2011). However, high affect spin appears to be related to instability in interpersonal relationships (Timmermans et al., 2010), failure to receive positive feedback from others (Côté et al., 2006), and depression (Kuppens et al., 2007), which may weaken career exploration and self-efficacy in career decision-making. Consequently, high affect spin would lower the facilitative effect of proactive personality on career maturity.

### Theoretical and practical implications

This study contributes to theoretical perspectives on career decision-making processes and emphasizes the moderating role of affect spin. A prior study (Jung et al., 2015) found that affect spin moderates the relationship between future time perspective and career decision-making self-efficacy, which in turn influences career choice anxiety. However, the present study investigated affect spin's moderating effect on the relationships between proactive personality, career indecision, and career maturity. These results extend Jung et al.'s (2015) findings, particularly when considering the fact that future time perspective is a state-like variable and proactive personality is a trait-like variable. Thus, affect spin would function as a strong link (i.e., as a moderator) between personality variables—including both state- and trait-like—and career decision-making processes. Additionally, the DRM used in this study improves upon the methodology for investigating affect spin by overcoming the ESM limitations arising from a short data collection period that could lead to biased results (Kuppens et al., 2007; Jung et al., 2015). In the DRM, participants evaluate their daily affective experiences for 21 days, which averages out the variability in affective events experienced. Furthermore, this study is the first to investigate the facilitative role of proactive personality in college students' career indecision and career maturity. According to the present results, highly proactive individuals would show low career indecision and high career maturity.

This study has practical implications for both researchers and college counselors. Researchers face difficulties in using the ESM and DRM because participants often find that rating their affect several times for 7 days or 3 weeks is burdensome. Studies on affect spin among employees should provide monetary compensation to participants. For instance, Beal et al. (2013) paid $50 per person to encourage participation in an affect spin study. A valid and reliable scale that measures affect spin needs to be developed to improve data collection methods.

Eid and Diener (1999) indicated that affect variability is considered a distinctive aspect of the personality. Further, Kuppens et al. (2007) suggested that individual differences in affect variability persist over time (Larsen, 1987; Penner et al., 1994; Eaton and Funder, 2001). Given these theoretical assumptions, developing an affect spin scale may be feasible. Counselors can help students make decisions about their future careers by facilitating a proactive personality and reducing affect spin. The former is especially difficult because of its trait-like nature. However, since proactive motivation is positively associated with career decidedness, counselors can motivate students to engage in proactive career behaviors (Hirschi et al., 2013). Additionally, empowerment programs, which are positively associated with proactive personality, might enhance proactivity in less proactive students (Judge and Ilies, 2002). Furthermore, practitioners can conduct interventions to regulate emotions in order to lessen affect spin. For example, mindfulness interventions can be effective for students with high affect spin because it has been shown to reduce emotional reactivity when experiencing negative affect. Britton et al. (2012) showed that participants of mindfulness interventions reported less anxiety during a stress test.

### Limitations and future directions

The current study is not without its limitations. First, it used a single-administration survey design to investigate the role of affect spin in the relationships between proactive personality, career indecision, and career maturity; this could result in common method bias, thereby reducing the validity of the conclusions (Podsakoff et al., 2003). To compensate for this bias, future studies should be designed and conducted using multiple data collection methods. Second, future research should include longitudinal data or experimental approaches to ascertain the causal relationships between proactive personality, career indecision, and career maturity. Third, the author focused on career decision processes such as career indecision and career maturity as outcome variables. However, these two variable were highly correlated each other in the present study (*r* = −0.85, *p* < 0.01). As such, although both a prior study (Brusoski et al., 1993) and the present one considered career indecision and career maturity as separated constructs, it may be redundant to include both of them as outcome variables. Finally, although this study controlled for valence and activation variability, affective home base—defined as a baseline affective state around which affect fluctuates (Kuppens et al., 2010)—was not considered. Future studies should control for affective home base as this may be related to career decisions by reflecting individual differences.

## Conclusion

The present study is the first to examine the moderating effect of affect spin on the associations between proactive personality, career indecision, and career maturity. Specifically, individual differences in affect spin seem to play an important role in moderating the effects of proactive personality on career indecision and career maturity. These results suggest that enhancing college students' proactive personality and reducing their affect spin can foster better career decision-making.

### Conflict of interest statement

The author declares that the research was conducted in the absence of any commercial or financial relationships that could be construed as a potential conflict of interest.
